# Cystinuria in an Australian Cattle Dog Family—A Seemingly Androgen-Associated Autosomal Dominant Trait

**DOI:** 10.3390/vetsci13010111

**Published:** 2026-01-22

**Authors:** Alexandra Kehl, Maria Brockmann, Sabine Helmes, Andrea Hildebrand, Sabine Döll, Elisabeth Mueller, Urs Giger

**Affiliations:** 1 Laboklin GmbH & Co. KG, 97688 Bad Kissingen, Germany; brockmann@laboklin.com (M.B.);; 2 Comparative Experimental Pathology, School of Medicine, Technical University of Munich (TUM), 80333 Munich, Germany; 3 Kleintierpraxis Helmes, 53347 Alfter, Germany; 4 Australian Cattle Dog Club Deutschland e.V., 97490 Maibach, Germany; 5 Vetsuisse Faculty, University of Zürich, 8057 Zürich, Switzerland; giger@vet.upenn.edu; 6 School of Veterinary Medicine, University of Pennsylvania, Philadelphia, PA 19104, USA

**Keywords:** canine, *SLC3A1*, sex, cystine, cystine urolithiasis, COLA, renal tubular defect

## Abstract

In Australian Cattle Dogs (AUCDs), cystinuria was reported to be an autosomal dominant trait caused by a 6 bp deletion in the *SLC3A1* gene (type II-A). Here we report an androgen association in this breed. A family of 11 adult AUCDs (five intact and one neutered male and five females) was genotyped for the *SLC3A1* c.1095_1100del variant, and urine was examined for concentrations of cystine, ornithine, lysine, and arginine (COLA). Among this family, three males and five females tested heterozygous for the mutation, while all other AUCDs were homozygous for the wild-type allele. The two heterozygous intact males had severe COLA-uria, which decreased markedly after castration. Neither the third heterozygous male with a history of cystine calculi but already castrated nor the five heterozygous females exhibited increased COLA-uria. Heterozygosity for the 6 bp deletion in the *SLC3A1* gene was associated with cystinuria in intact male AUCDs, but not in females. Castration of the heterozygous males reduced the cystinuria. Either the type II-A cystinuria in the AUCDs is an androgen-associated dominant trait, or this family also has another type III (androgen-dependent) cystinuria. Larger surveys are needed to further define the type(s) of cystinuria and effects of castration in AUCDs.

## Simple Summary

Amino acid transporter defects in the kidney can lead to cystinuria. As cystine is poorly soluble, excessive urinary excretion can lead to the formation of cystine crystals and calculi. In Australian Cattle Dogs (AUCDs), a mutation in the *SLC3A1* gene was described to cause an autosomal dominant trait (cystinuria type II-A). Here we report on a family of AUCDs, where in cystinuric males heterozygous for the known mutation cystinuria decreased markedly with castration. This suggests that cystinuria and cystine calculi formation in males heterozygous for the *SLC3A1* gene mutation are androgen-associated.

## 1. Introduction

Cystinuria was first reported nearly a century ago as one of the first inborn errors of metabolism in dogs [1]. It is now recognized as a heterogeneous hereditary kidney disorder [1,2,3,4,5]. Instead of the normal >99% reabsorption of cystine, ornithine, lysine, and arginine (COLA) in the proximal renal tubules, these specific amino acids are lost in urine, resulting in increased COLA-uria [6,7]. Because cystine readily precipitates at higher concentrations in slightly acidic urine, this renal reabsorption defect can lead to urinary cystine crystals and calculi formation and clinically to pollakisuria, stranguria, hematuria and even urinary tract obstruction [6,7,8]. Mainly, intact male dogs are metabolically and clinically affected [9,10]. The loss of the other three amino acids ornithine, lysine, and arginine does not cause clinical signs, hence the name cystinuria.

The rBAT and b^0,+^AT subunits of the COLA transporter in the proximal renal tubules are encoded by the *SLC3A1* and *SLC7A9* genes, respectively [2,4,7]. Although both genes have been sequenced across many canine breeds, only a few pathogenic variants have been identified to date, each confined to a small number of specific breeds and currently detectable through diagnostic testing. Specifically known are type I-A cystinuria, type II-A cystinuria, and type II-B cystinuria. In type I-A cystinuria, two premature stop codons in the *SLC3A1* gene cause autosomal recessive traits in Newfoundland and Labrador retriever dogs, respectively. Homozygous dogs of both genders remain cystinuric following neutering [2,4,5,8,11]. In type II-A cystinuria, a 6 bp deletion in the *SLC3A1* gene causes an autosomal dominant trait in AUCDs [5]; this variant is the topic of the current report. In type II-B cystinuria, a single amino acid variant in the *SLC7A9* gene causes an autosomal dominant trait in Miniature Pinschers, but it occurs rarely [5].

While the above specific gene variants have been associated with cystinuria in both genders, males more commonly develop and are obstructed by cystine calculi [4,5,8,11].

Additionally, another type of cystinuria, previously referred to as non-type I cystinuria and reclassified as type III in 2013 [5,12] has been diagnosed in various breeds [5,11,12,13,14,15,16]. Cystinuria in these breeds is considered androgen-dependent [2,11,12,17], consequently, castration of affected males eliminates their cystinuria and COLA-uria, effectively resulting in clinical cure [12,17,18].

In breeds with cystinuria classified as type III, intact adult cystinuric males have less severe COLA-uria and develop cystine urolithiasis later in life compared to the above described breeds with type I and II cystinuria [2,5,11]. However, only one non-deleterious single amino acid exchange in rBAT has been associated with androgen-dependent cystinuria (type III cystinuria) in a few breeds, mostly in Mastiffs and French and English bulldogs [2,17,19]. Thus, this has been considered to be only an *SLC3A1* gene marker, while the molecular genetic basis and pathophysiology remain to be elucidated.

We report here on genotyping and COLA results in an AUCD family with cystinuria, indicating the presence of a dominant variant together with the unique new observation of additional androgen association in male AUCDs heterozygous for the 6 bp deletion in the *SLC3A1* gene.

## 2. Materials and Methods

After the discovery of a male AUCD with cystine urolithiasis (Dog 1), voided free-catch urine and blood or cheek swab samples from this and related dogs registered by the Australian Cattle Dog Club Deutschland (ACDCD) were submitted by AUCD breeders and owners for routine clinical diagnostic testing to Laboklin GmbH & Co. KG (Bad Kissingen, Germany) between September and November 2024. Neutering of two cystinuric males took place shortly thereafter. Follow-up urine samples from these two males were taken in 2025, eight resp. twelve months after neutering.

Urinary amino acids were analyzed by Liquid Chromatography–Tandem Mass Spectrometry (LC-MS/MS). An Acquity UPLC I Class Plus coupled to a Waters Xevo TQ XS mass spectrometer (Waters, Eschborn, Germany) was used to determine cystine, ornithine, lysine, and arginine concentrations in µmol/gram creatinine. Urinary creatinine concentrations were measured using a routine diagnostic test (Cobas 8000, Roche Diagnostics GmbH, Mannheim, Germany).

For genotyping, genomic DNA was isolated from blood or cheek swabs and amplified with a FAM-labeled forward primer and an unmarked reverse primer spanning the 6 bp deletion in the *SLC3A1* gene. Fragment length polymorphism between the mutant and wild-type alleles was analyzed using an ABI 3730XL Genetic Analyser and GeneMapper Software 5 (Thermo Fisher Scientific, Darmstadt, Germany).

## 3. Results

**Figure 1:**
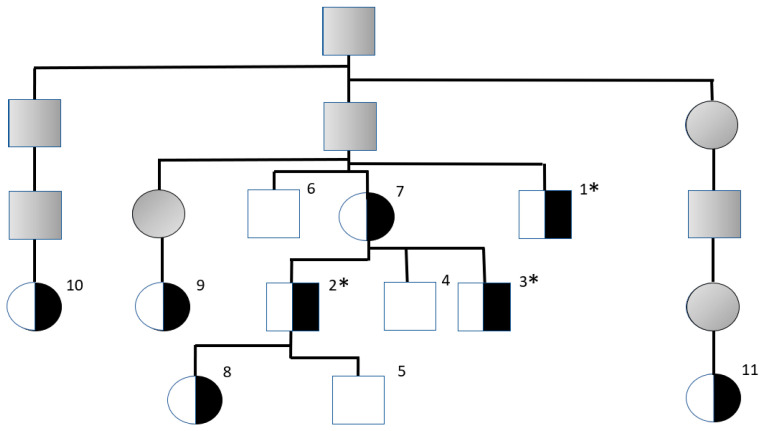
Pedigree of the Australian Cattle Dog family with cystinuria and *SLC3A1* genotyping results. Male and female dogs were heterozygous for the 6 bp deletion in the *SLC3A1* gene, but only the males were cystinuric (*). Squares and circles represent males and females, respectively. Half-filled symbols indicate heterozygous individuals. Shaded symbols refer to individuals that were not tested. Numbers refer to dog numbers in Table 1.

This survey of an AUCD family was initiated after a male AUCD (Dog 1) was found to have cystine urolithiasis. The eleven AUCDs studied (Figure 1 and Table 1) were related to a popular sire suspected of having several cystinuric offspring in its pedigree. Samples for initial testing were received between September and November 2024 from six males and five females; all of them were adults (Table 1). This included Dog 1, which had previously been diagnosed with cystine urolithiasis but was only COLA tested after he was already castrated. None of the other dogs in the family had been clinically reported to have developed cystine calculi or urinary tract obstruction.

Urinary sediment evaluations did not reveal cystine crystals in any of the dogs. However, two intact male dogs had very high urinary cystine (>1000 µmol/g creatinine) and COLA (>2000 µmol/g creatinine) concentrations, thereby showing cystinuria. Interestingly, urinary cystine and COLA concentrations normalized or decreased markedly in all three male AUCDs with evidence of cystinuria after castration (Table 1).

The three males with evidence of cystine calculi or increased COLA-uria were heterozygous for the *SLC3A1* 6 bp deletion, while the other three non-cystinuric and non-COLA-uric males were homozygous for the wild-type allele. All five females were also heterozygous for the mutant *SLC3A1* variant reported in AUCDs but non-cystinuric and non-COLA-uric. Notably, in this genotyped family, no AUCDs were homozygous for the 6 bp deletion in the *SLC3A1* gene.

## 4. Discussion

This exploratory observational study indicates the presence of androgen-associated cystinuria in male AUCDs heterozygous for the 6 bp deletion in the *SLC3A1* gene, thus expanding the understanding of cystinuria in heterozygous male AUCDs.

In 2013, a 6 bp deletion in the *SLC3A1* gene, predicted to delete two out of three adjacent threonines in rBAT and thus being considered deleterious, was discovered in six cystinuric AUCDs [5]. In that study, three males and one female were homozygous, and two males were heterozygous for this breed-specific *SLC3A1* variant [5]. The homozygous males developed cystine uroliths before one year of age, while the homozygous female and the two heterozygous males were cystinuric but did not have clinically detected cystine calculi until four to five years of age [5]. Urinary COLA concentrations, when measured, were increased and higher in AUCDs homozygous versus heterozygous for the variant [5]. These findings are consistent with an autosomal dominant trait, and thus cystinuria in AUCDs was classified as type II-A cystinuria [5]. However, the potential effects of castration were not examined in that original study; specifically, no urinary COLA values were reported for the castrated heterozygous male described. The time interval between castration and determination of urinary COLA values in homozygous males was not reported. In a more recent survey including AUCDs with cystine calculi, the time interval between castration and determination of urinary COLA values was at least three months, but no genotyping information was provided for the clinically affected neutered males [11].

In the present family survey of eleven related AUCDs conducted in late 2024, three males, but none of the five females, heterozygous for the 6 bp deletion in the *SLC3A1* gene, exhibited cystine urolithiasis and/or increased COLA-uria. No cystine crystals were detected. However, the inconsistent presence of these was noted previously [21]. Upon castration of these three affected heterozygous males, the severe COLA-uria normalized or decreased markedly, and no clinical cystine calculi formation was reported thereafter in any of these dogs. These unexpected findings suggest that the *SLC3A1* variant is androgen-associated in at least heterozygous male AUCDs. The underlying molecular mechanisms do not appear to be equivalent to those of the already known type III cystinuria. Androgen association observed in AUCDs may rather represent a modifying effect. However, our study is based on a single family (*n* = 11), which limits the generalizability of the findings.

Unfortunately, this survey lacks any AUCDs homozygous for the 6 bp deletion in the *SLC3A1* gene. While male or female AUCDs homozygous for the deletion were reported to have severe COLA-uria in the original report [5], the effect of neutering was not assessed. Due to the lack of any AUCDs homozygous for the mutation in this study, it remains unknown whether cystinuric AUCDs homozygous for the 6 bp deletion will also show markedly reduced urinary COLA concentrations after castration, thereby eliminating their risk of further cystine stone formation and resulting in clinical cure.

Heterozygous females had no elevated COLA-uria or clinical signs of cystine calculi formation in this AUCD family. In the original report, there was one homozygous female AUCD with cystinuria [5]. However, in the current survey, no AUCDs were homozygous for this deletion. Therefore, it was not possible to make any claims on the effect of castration in homozygous AUCDs. Moreover, this was a small survey of related AUCD dogs, and it is possible that, in addition to the 6 bp deletion in the *SLC3A1* gene, other genes and metabolic derangements yet to be determined are responsible for this apparent androgen association. While genotyping for the *SLC3A1* variant in AUCDs remains warranted, castration of at least heterozygous males appears to prevent the development of marked cystinuria, effectively providing clinical cure and preventing their use in breeding. However, additional studies are required to determine whether the *SLC3A1* 6 bp deletion is directly influenced by androgens. So far, to the knowledge of the authors, neither in this study nor in any other study reporting canine cystinuria androgen hormones were determined.

In conclusion, the previously reported 6 bp deletion in the *SLC3A1* gene was confirmed to be a dominant variant that causes cystinuria in intact male AUCDs (heterozygous males were cystinuric). In this family, the variant appears to be androgen-associated based on the fact that the cystinuria in heterozygous AUCD males decreased markedly with castration, and heterozygous females were not cystinuric.

## Figures and Tables

**Table 1:** Results of *SLC3A1* c.1095_1100del genotyping and urinary cystine and COLA concentrations in a family of Australian Cattle Dogs.

Dog	Sex	Genotype	Urinary Cystine µmol/g Creatinine		Urinary COLA µmol/g Creatinine	
			Intact	Neutered	Intact	Neutered
1	M *	w/cy	ND	68	ND	196
2	M	w/cy	**>1500 ^#^**	140	**>5630**	233
3	M	w/cy	**1181**	**244**	**2801**	410
4	M	w/w	21		119	
5	M	w/w	8		211	
6	M	w/w	1		88	
7	F	w/cy	53		157	
8	F	w/cy	69		602	
9	F	w/cy	47		144	
10	F	w/cy	23		114	
11	F	w/cy	23		100	

M, male; F, female; ND, not determined. cy, mutant allele; w, wild-type; *, diagnosed previously with cystine urolithiasis; Urinary concentrations of >200 µmol cystine and >700 µmol COLA/g creatinine were considered to indicate cystinuria [17,20]. ^#^ >1500 µmol cystine/gram creatinine was the laboratory’s upper limit of measurement. Abnormal results are shown in bold.

## Data Availability

The original contributions presented in this study are included in the article. Further inquiries can be directed to the corresponding author.
